# A clinical trial evaluating feasibility and acceptability of a brain-computer interface for telerehabilitation in patients with stroke

**DOI:** 10.1186/s12984-025-01607-x

**Published:** 2025-04-24

**Authors:** Salem Mansour, Joshua Giles, Krishnan P. S. Nair, Rebecca Marshall, Ali Ali, Mahnaz Arvaneh

**Affiliations:** 1 Department of Automatic Control and Systems Engineering, University of Sheffield, Sheffield, UK; 2 Department of Neurology, Sheffield Teaching Hospitals NHS Foundation Trust, Sheffield, UK; 3 Neuroscience Institute, University of Sheffield, Sheffield, UK

## Abstract

**Background:**

We have created a groundbreaking telerehabilitation system known as Tele BCI-FES. This innovative system merges brain-computer interface (BCI) and functional electrical stimulation (FES) technologies to rehabilitate upper limb function following a stroke. Our system pioneers the concept of allowing patients to undergo BCI therapy from the comfort of their homes, while ensuring supervised therapy and real-time adjustment capabilities. In this paper, we introduce our single-arm clinical trial, which evaluates the feasibility and acceptance of this proposed system as a telerehabilitation solution for upper extremity recovery in stroke survivors.

**Method:**

The study involved eight chronic patients with stroke and their caregivers who were recruited to attend nine home-based Tele BCI-FES sessions (three sessions per week) while receiving remote support from the research team. The primary outcomes of this study were recruitment and retention rates, as well as participants perception on the adoption of technology. The secondary outcomes involved assessing improvements in upper extremity function using the Fugl-Meyer Assessment for Upper Extremity (FMA_UE) and the Leeds Arm Spasticity Impact Scale.

**Results:**

Seven chronic patients with stroke completed the home-based Tele BCI-FES sessions, with high retention (87.5%) and recruitment rates (86.7%). Although participants provided mixed feedback on setup ease, they found the system progressively easier to use, and the setup process became more efficient with continued sessions. Participants suggested modifications to enhance user experience. Following the intervention, a significant increase in FMA_UE scores was observed, with an average improvement of 3.83 points (p = 0.032). The observed improvement of 3.83 points in the FMA-UE score approaches the reported Minimal clinically important difference of 4.25 points for patients with chronic stroke.

**Conclusion:**

This study serves as a proof of concept, showcasing the feasibility and acceptability of the proposed Tele BCI-FES system for rehabilitating the upper extremities of stroke survivors. While some participants demonstrated significant improvements in FMA-UE scores, these findings are not generalizable, as they were derived from a small-scale feasibility study. The results should be interpreted cautiously within the study’s specific context. Additionally, the intervention was not compared to other therapeutic approaches, limiting conclusions regarding its relative effectiveness. To further validate the efficacy of the proposed Tele BCI-FES system, it is essential to conduct additional research with larger sample sizes and extended rehabilitation sessions. Moreover, future studies should include comparisons with other therapeutic approaches to better evaluate the relative effectiveness of this intervention.

*Trial registration* This clinical study is registered at clinicaltrials.gov https://clinicaltrials.gov/study/NCT05215522 under the study identifier (NCT05215522) and registered with the ISRCTN registry https://doi.org/10.1186/ISRCTN42991002 (ISRCTN42991002).

## Introduction

Among 1.2 million stroke survivors in the UK, 77% experience upper limb weakness, of which 66% experience weakness beyond 6 months [1]. Upper limb motor impairments are common among stroke survivors and are associated with an increased risk of falling, dependency on care, and reduced quality of life [2, 3]. The annual financial burden of the stroke in the UK is around £25.6 bn, and that amount is predicted to increase significantly over the next 20 years [3, 4]. Therefore, there is an urgent need to develop more effective and efficient rehabilitation techniques in order to reduce the disabling effects of stroke.

Currently, available rehabilitation methods focus on assisting in recovery within the first few months after the stroke. These methods include the use of constraint-induced movement therapy and functional electrical stimulation (FES)-based therapies as well as robotic based therapies [5, 6]. These therapies require intensive intervention from therapists, and/or are passive, requiring limited effort from the patient themselves.

Brain-computer interfaces (BCIs) can enhance existing therapies by actively involving patients, enabling them to control the movement of their impaired limb through their own thoughts [7]. This active participation enhances neuroplasticity in patients with stroke. Our recent meta-analysis demonstrated superior efficacy of BCI in improving upper limb motor function for both patients in the subacute and chronic phase compared to conventional therapies such as constraint-induced movement therapy and passive FES interventions. These comparisons accounted for similar treatment durations and intensity [7].

Although the results from these studies are promising there are still a number of limitations with the technology. One primary issue is that the equipment used for BCI based rehabilitation is bulky, expensive, technically complex, and requires careful placement of numerous electrodes. As a result, the BCI based rehabilitation process is currently limited to hospitals or labs due to these hardware constraints, which can also create additional challenges. The need to travel frequently to the hospital for receiving rehabilitation can be challenging for patients with stroke with mobility problems. Another issue that limits the real-world application of this technology is the calibration time required by a BCI for training before each use [8]. In some cases, it can take up to 20 min to calibrate the BCI before rehabilitation starts [9].

Therefore, the objective of this study is to develop a novel BCI system that is both feasible and appealing for stroke survivors to utilize in home-based rehabilitation. To achieve this, we have developed a novel portable BCI system specifically designed for stroke rehabilitation. This system enables patients to conveniently use it in the comfort of their own homes while receiving remote supervision through the internet when required.

Several countries, including the United Kingdom, are experiencing a significant shortage of physiotherapists and occupational therapists [10]. With an aging population and increasing demand for rehabilitation services, home-based approaches like Tele BCI-FES could potentially serve as viable alternatives to traditional methods that heavily depend on health care professional involvement. If accepted by patients, Tele BCI-FES can offer the advantage of enabling an allied health professional to remotely supervise multiple sessions simultaneously, regardless of geographical location. However, a comprehensive health-economics analysis is essential to further evaluate the cost-effectiveness of technology-driven home-based rehabilitation approaches. Our BCI system classifies the EEG signals collected from the patient and identifies when the patient is attempting to move their weakened hand or staying still. When the BCI detects EEG signals associated with attempted movement, it activates a functional electrical stimulation system to provide assistance with the movement.

In short, the study objectives are:

- To assess if the patients can use our Tele BCI-FES system at home for post-stroke upper limb rehabilitation.
- To assess the patient’s perspective about the use of our Tele BCI-FES device for home-based arm rehabilitation

We will use the data from this study to improve the design of the Tele BCI-FES system and conduct a larger clinical study.

## Materials and methods

### Tele BCI-FES system design

**Fig. 1:**
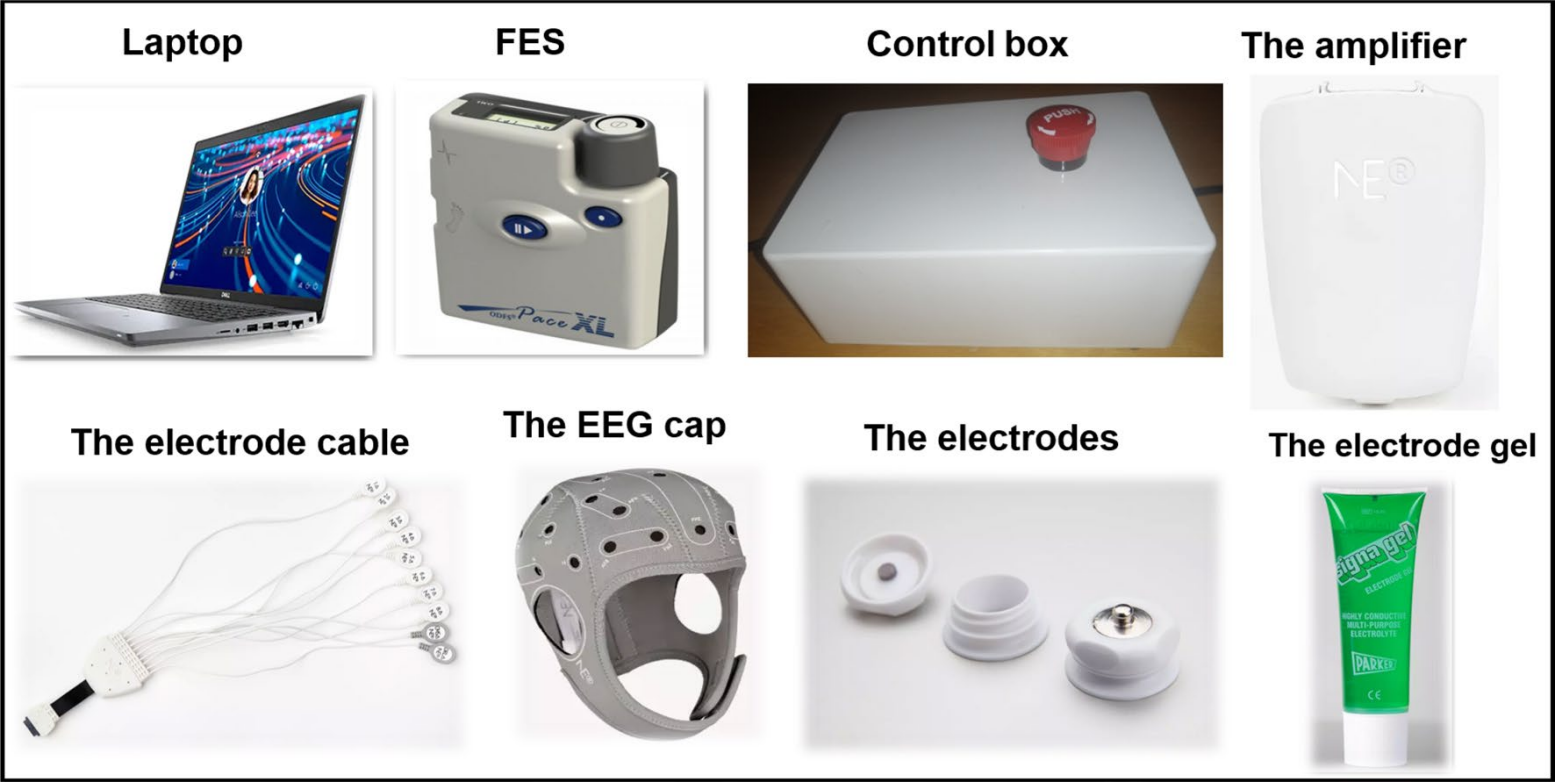
This figure shows the Tele BCI-FES equipment that the participants used at home which included a latitude 5420 dell laptop, an Odstock ODFS® Pace XL FES unit, a control box, an ENOBIO8 EEG amplifier with Electrode lead, EEG cap with electrodes and a bottle of electrode gel used during the study

**Fig. 2:**
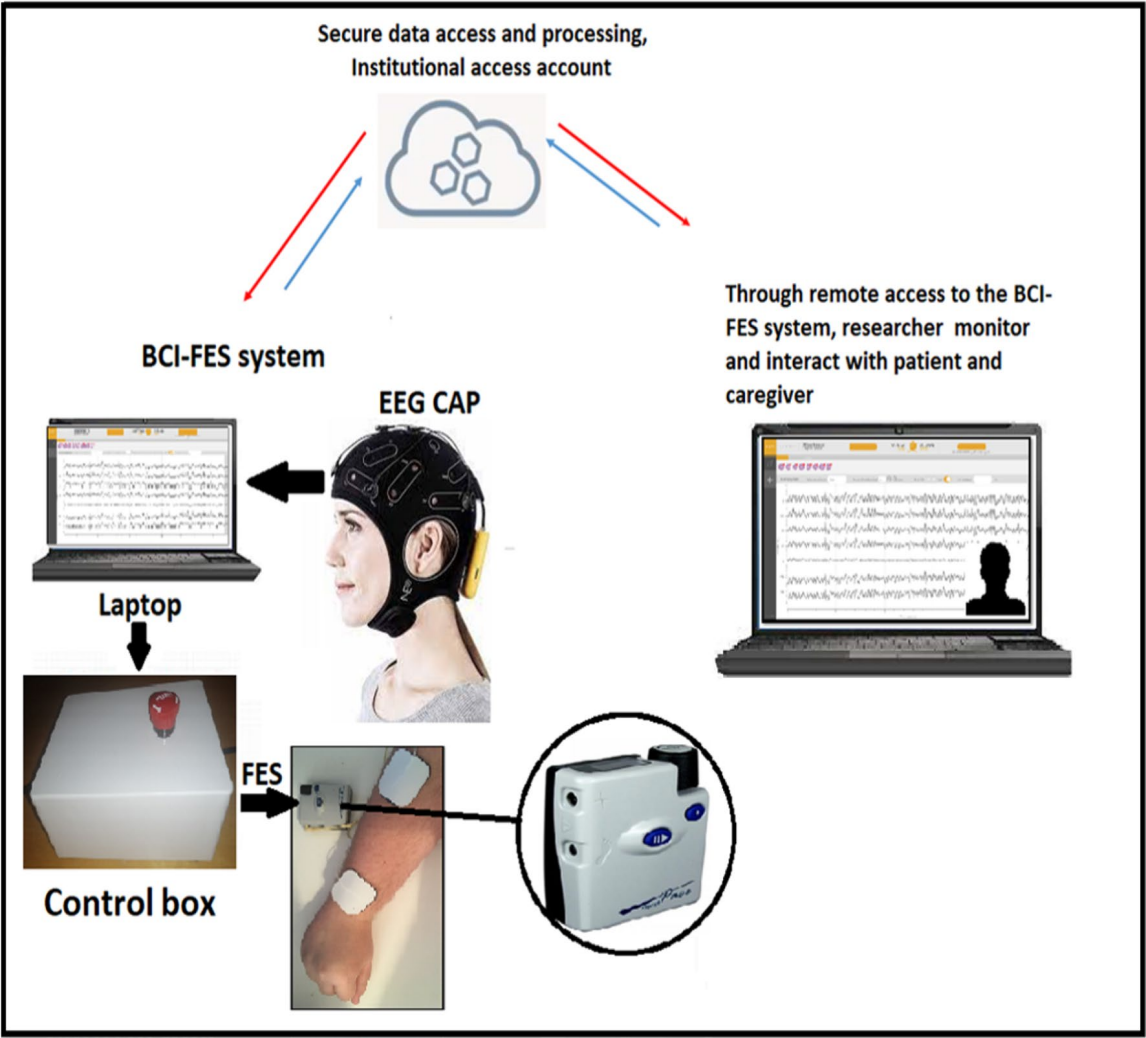
The proposed Tele BCI-FES system for upper-limb stroke rehabilitation. The control box is equipped with an emergency button that instantly halts the system in case of any emergencies. Additionally, an Arduino board is used in the control box to receive commands from the laptop and send them to activate the FES device

To complete this study it was necessary to create a novel system of hardware and software that was portable and easy to set up so that the participants could set it up at their homes. We made all attempts to ensure that the device is very user friendly. We conducted multiple patient public involvement (PPI) sessions with individuals undergoing upper limb rehabilitation after a stroke. These sessions were aimed at gathering valuable feedback and suggestions from the patients themselves regarding the necessary improvements for the system. The final Tele BCI-FES components are shown in Figs. 1 and 2.

During the experiment, a Dell laptop model Latitude 5420 was used as the platform for presenting instructions and providing feedback to the participant. The laptop had remote access and remote control computer software installed, which enabled the patient to communicate with the physiotherapist and/or researcher during the rehabilitation session. This software also allowed the researcher and/or physiotherapist to monitor the quality of the signals recorded from the Tele BCI-FES system and make necessary adjustments to its parameters. Common adjustments included providing guidance to patients and caregivers on applying additional gel to specific electrodes to improve conductivity, recalibrating the BCI system to account for variations in signal quality, and modifying the FES intensity to ensure patient comfort and optimized stimulation. Furthermore, the laptop was utilized for preprocessing and classifying the EEG signals collected by the EEG system. The selected EEG system for data collection was the Neuroelectrics ENOBIO 8, which captured signals from eight channels using gel-filled electrodes that were secured within a cap. This EEG system was selected for its compact size, ease of set up and adaptability, with the location of the electrodes being personalized for each of the participants. The FES device was the Odstock OML XL pace unit which is currently used by the NHS England and is recommended by the National Institute for Health and Care Excellence (NICE). To facilitate communication between the laptop and the FES stimulator, a control box was designed and created. The control box incorporates an Arduino programmed to replicate the signal typically transmitted to the FES through a foot switch. By replicating this signal, the laptop can safely activate the FES.

### Tele BCI-FES single-arm clinical trial design

#### Inclusion and exclusion criteria

The study involved participants aged 18 and older who had experienced an ischemic or hemorrhagic stroke at least 6 months ago. These participants had residual arm weakness resulting from the stroke, affecting their ability to perform daily activities. Other inclusion criteria were a Fugl-Meyer score of upper limb less than 45, cognitive and language abilities to understand and participate in the study protocol, and having a caregiver who is willing to help deliver the Tele BCI-FES intervention. Furthermore, we included only participants who could remain seated for 1 h with or without support, and were able to give consent and understand instructions.

The exclusion criteria for selecting participants were as follows: cognitive impairment that would interfere with the ability to comply with the experimental protocol or provide informed consent; dermatological, rheumatologic or orthopaedic illnesses of the affected arm interfering with movement of the elbow, history of epilepsy, having pacemaker or any other electrical implanted devices, pregnancy, severe dystonia/spasm, pre-existing severe systemic disorders such as cardiovascular disease, active cancer or renal disease, end stage pulmonary or cardiovascular disease and psychiatric illness including severe alcohol or drug abuse, and severe tactile hypersensitivity. Participants who were unable to complete the baseline assessments or whose BCI accuracy during the calibration session fell below the chance level (58%) were excluded from the study. This threshold was determined using the inverse binomial distribution function, which indicates that a 99% confidence level for chance performance in 110 calibration BCI trials corresponds to approximately 58%. As a result, participants with calibration session BCI accuracy below 58% were considered to be performing at chance level and excluded from the study [11]. Participants were also excluded if they previously participated in other upper limb rehabilitation studies.

#### Initial assessment session

The clinical team distributed the patient information sheet to patients with stroke attending the outpatient or Functional Electrical Stimulation clinic at Sheffield Teaching Hospitals. Patients who expressed interest in participating and provided consent underwent an eligibility screening process. Eligible patients were then invited to the University of Sheffield for their initial visit, where their eligibility was reassessed and functional assessments were conducted. The optimal electrode location and stimulation intensity for the FES were determined for each participant. Finally, the BCI system was explained to them, and a calibration session with the BCI system was conducted.

***BCI calibration***

**Fig. 3:**
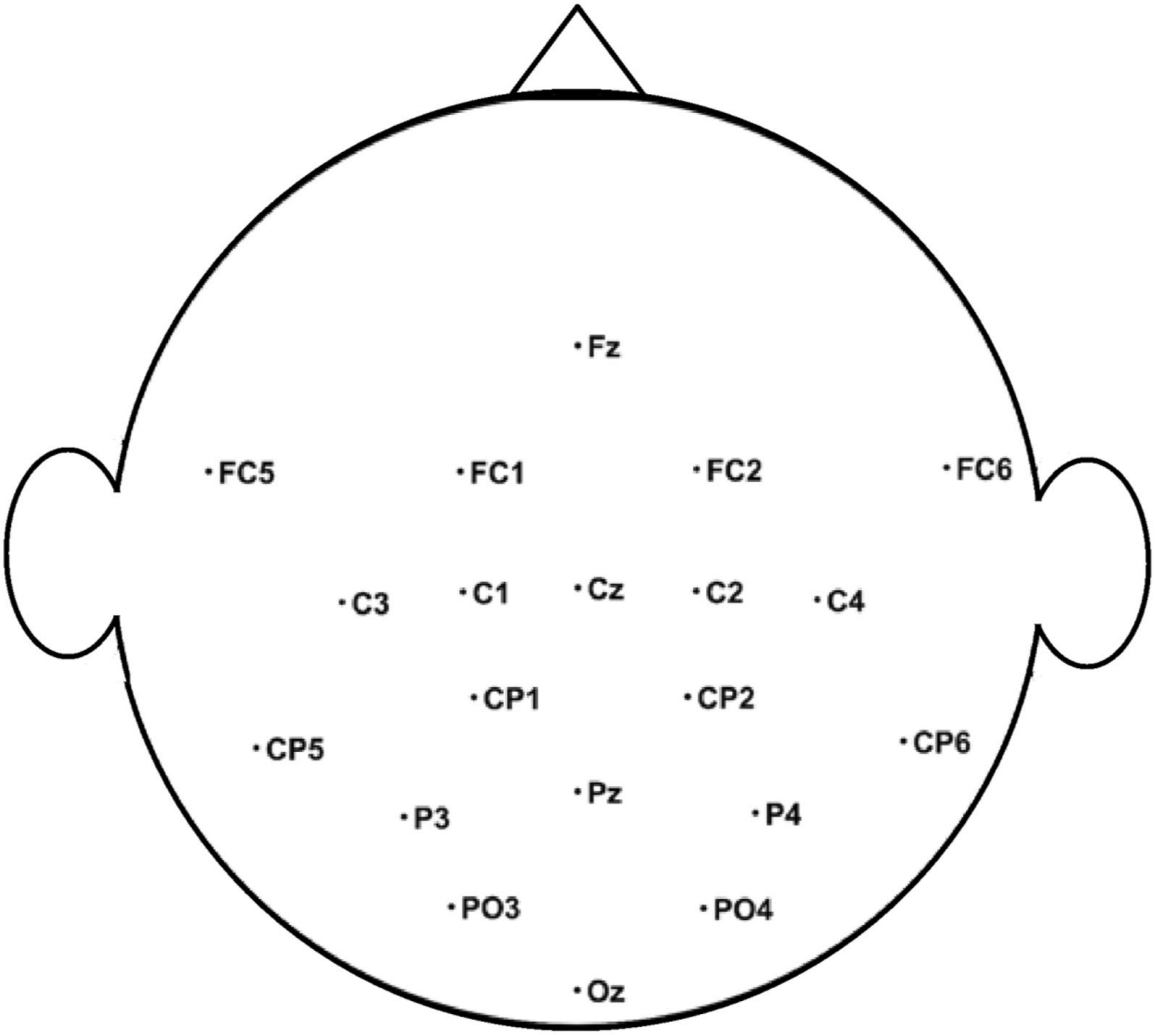
The figure shows the position of 20 channels that were used for BCI calibration session

In the BCI calibration session, participants were instructed on how to set up and clean the EEG system. In addition, EEG signals were collected from the participants to assess the system’s accuracy. During BCI calibration, 20 channels were used to collect EEG signals, as shown in Fig. 3.

**Fig. 4:**
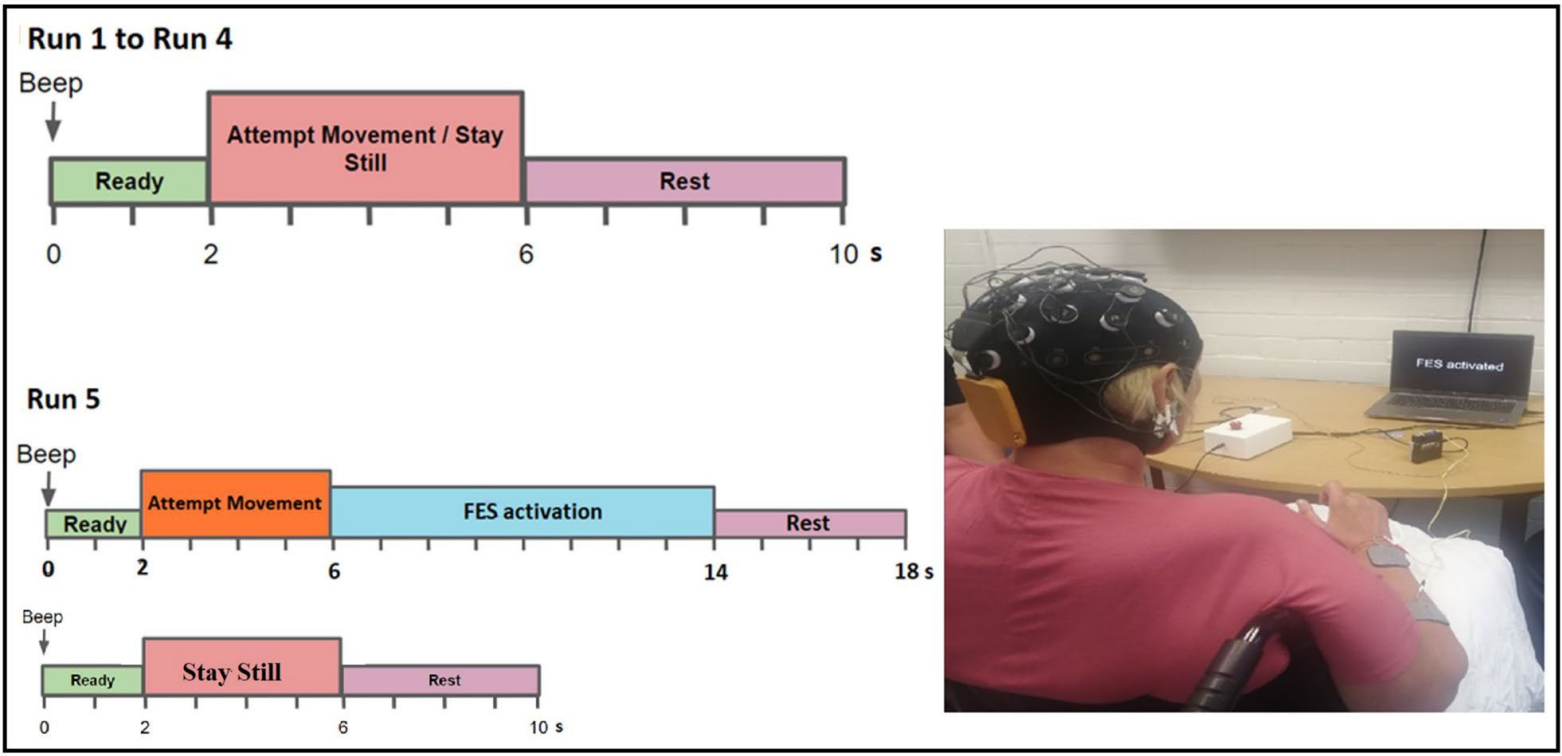
Timing of the trials for the 5 runs of the BCI calibration session. The FES activates to produce hand movement when the participant is instructed to try to move their weakened hand during the final run (i.e, run 5)

The participants were instructed to attempt to extend their weakened hand with their fingers and wrist upwards so that the palm was facing forward and the fingers upwards. Those unable to produce any movement were asked to try to focus on this movement and imagine their hand moving. The BCI calibration session consisted of 5 runs, where each run had 11 trials of the attempt movement task and 11 trials of the staying still in random order. As shown in Fig. 4, for runs one to four each trial lasted 10 s, consisting of a two-second ready period following a beep, four seconds of either attempted movement or staying still, and four seconds of rest. On the fifth run, the FES was activated for the trials that the participants attempted to move their weakened hands, increasing the trial length to 18 s. Indeed, the fifth run gave the participants the chance to familiarize themselves with the FES activation. After each run, a break was given to the participants. On average, the BCI calibration session lasted about an hour, including the cap set up, demonstration of the equipment, collection of the EEG, and breaks.

After the EEG was collected it was used to train the BCI model and evaluate the participant’s ability to control the BCI. The extracted EEG data were filtered using a zero-phase band-pass filter from 8 to 13 Hz. Then the BCI features were extracted using a common spatial patterns (CSP) algorithm. Next, the extracted features were classified using a linear discriminant analysis (LDA) classier. The classifier outcomes were objectively evaluated using 10 runs $\times$ 10-fold cross-validation.

Following the classification of the EEG data from 20 channels, the best eight electrodes were obtained. Participants with a BCI classification accuracy greater than 58% were then offered a 3-week home-based rehabilitation using Tele BCI-FES system.

#### Home sessions

Enrolled participants were provided with a Tele BCI-FES system at the end of the screening session to take home with them. This kit included all the BCI and FES equipment required to conduct the intervention at home, as shown in Fig. 1. In addition to the equipment, the participant was given instructions on the set-up of the EEG and FES, investigator contact details, a remote meeting schedule, and a custom EEG electrode location map.

Throughout this study, 10 remote sessions, each lasting one hour were scheduled. The first one was for practicing and making sure the participant and their caregiver are comfortable in setting up and using the Tele BCI-FES system. The next 9 sessions (3 sessions per week) consisted of 10 min of preparation (instructions, setup/calibration time), 40 min of Tele BCI-FES rehabilitation, a 5 min patient interview on their experience with the session, and a 5 min interview on general health check and any adverse effects experienced before and within the session.

During the home sessions, participants, with the assistance of their carer and remote guidance from the researchers, completed the system setup, which involved the following steps:

- Powering on the laptop.
- Connecting the EEG amplifier and control box to the laptop.
- Connecting the FES to the control box.
- Applying the FES electrodes to the arm with the guidance of a remote physiotherapist.
- Placing the electrodes in the EEG cap.
- Applying the provided gel to the electrodes and wearing the EEG cap.

The setup process was initially demonstrated during the screening session. Once the laptop was turned on, the researchers/physiotherapist were available remotely to provide guidance and support with the setup. Using the Team Viewer, a remote access software, the researchers were able to remotely access and control the laptop to configure the necessary software and initiate a video call. Before proceeding with the Tele BCI-FES intervention, a brief checklist was completed to ensure the participant had not experienced any adverse reactions since the previous session and was comfortable continuing with the study.

After ensuring the proper setup and connection of the system, the participant engaged in a remote rehabilitation session under the remote supervision of the physiotherapist. During this session, the FES was activated by the BCI whenever an attempted movement was detected. The home rehabilitation session lasted approximately 45 min, consisting of five runs. Each run mirrored the structure of the fifth run from the screening session, followed by a break. At the end of each home session, the participant was asked to fill a brief quantitative questionnaire to report their perception of the Tele BCI-FES system at that session. Please see section for more details.

#### Final assessment session

After completing the home sessions, participants and their carers were invited to the University of Sheffield for a comprehensive post-assessment. This assessment included repeating the motor function evaluations conducted at screening to quantitatively measure the extent of hand function improvement achieved after the Tele-BCI-FES interventions. Following the post-assessment, in-depth qualitative interviews were conducted with participants and their carers. The interviews aimed to explore their experiences and perceptions regarding the use of the Tele-BCI-FES system.

### Primary outcomes

#### Recruitment and retention rates

Recruitment and retention rates were calculated to evaluate the success of the study in attracting and retaining participants [12]. The recruitment rate indicates the percentage of individuals who were approached to participate in the study and agreed to do so, while the retention rate represents the proportion of participants who completed the study in relation to the initial number of participants who enrolled. Study completion was considers as completing at least seven out of nine Tele BCI-FES home sessions.

#### Patients’ participation rate

Patients’ participation rate in the remote therapy sessions was assessed through the number of sessions they agreed to attend within a set period and using Pittsburgh Rehabilitation Participation Scale (PRPS) [13]. PRPS is scored on a 6-point scale that takes into account the patient’s engagement in therapy (1: none- patient refused entire session to 6: excellent- patient participated in all activities of the session). This score was provided by the researcher and physiotherapist at the end of each session.

#### Participants perception on adoption of technology

In order to evaluate the system’s feasibility and acceptability, cumulative questionnaires were collected after each session. A in-depth final questionnaire was conducted face to face when the participants returned to have their final functional assessment. The questionnaires specifically focused on the participants’ and carers’ experiences during the session, including the setup process, adherence to instructions, quality of supervision, and perceived effectiveness of the rehabilitation session. The participant and carer were asked to rate these experiences on a scale of 1–5 (where 1 is very difficult, 2 difficult, 3 normal, 4 easy, and 5 very easy), the patient answered the following questions:

1. How difficult or easy did the carer find the Tele BCI-FES equipment setup?
2. How difficult or easy was to communicate with the remote connection system?
3. How difficult or easy did you find the use of the Tele BCI-FES device for rehabilitation?
4. How easy or difficult did you find wearing the Tele BCI-FES equipment?

The participants were also asked if they would recommend the Tele BCI-FES system to other patients with stroke. In addition, they were asked whether there is anything about the Tele BCI-FES system that they believe needs to be improved.

### Secondary outcomes (functional assessment)

We conducted functional assessments both before and after the Tele BCI-FES intervention, using the upper extremity section of the Fugl-Meyer assessment (FMA_UE) [14]. This assessment assigns a numerical score to a patient’s motor function and can be used to measure changes in their motor function and to evaluate the effectiveness of the intervention. The FMA_UE score ranges from 0 to 66, with lower scores indicating greater impairment in upper limb function.

Additionally, we employed leeds arm spasticity impact scale (LASIS) to assess passive arm function in subjects who had spasticity and little to no active upper extremity movement [15, 16]. The LASIS consists of 12 items that assess passive and low-level active function. Items are evaluated from 0 to 4 (0 indicates no difficulty; 1 indicates slight difficulty; 2 indicates a moderate level of difficulty; 3 indicates extreme difficulty; and 4 indicates an inability to carry out the activity).

Finally, the numerical rating scale (NRS) was completed by each participant. On a scale of 0 (no pain) to 10 (severe pain), participants were asked to rate their level of pain using NRS scale [17].

### Statistical analysis

In this study, the Wilcoxon signed-rank test was applied as a nonparametric alternative to account for the small sample size [18]. This test was used to evaluate the significance of changes in outcome measures between the post-intervention and screening sessions. Data analysis was conducted using MATLAB, with a significance level set at $p=0.05$.

## Results

**Fig. 5:**
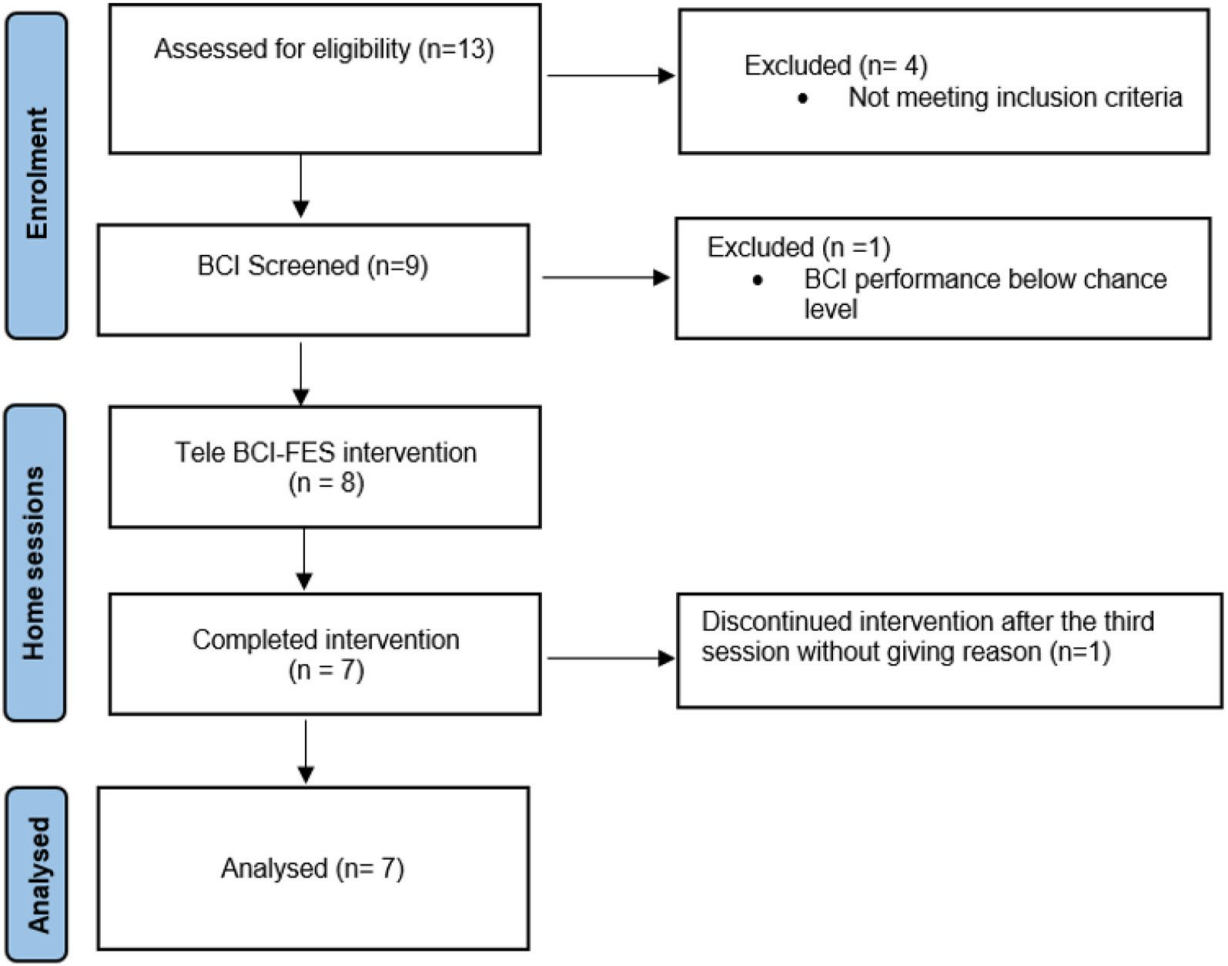
Flow chart of the study from enrollment to analysis

Figure 5 presents a flow chart of the Tele BCI-FES study, from enrollment to analysis.

### Participant characteristics

Nine participants attended the screening session and had their eligibility for participation assessed. Eight of these participants continued to complete the home sessions while one participant was excluded from the study because their BCI accuracy was below the chance level. Seven participants completed the study, while one participant decided to withdraw from the study after attending three sessions (see Fig. 5). The demographic information for each participant who participated in this study is shown in Table 1. One participant (P03) had to stop early after seven sessions due to health problems unrelated to the intervention and the final face-to-face session was delayed by three weeks due to illness. Another participant (P05) received botulinum toxin treatment before the start of the study and was therefore not included in the motor functional assessment as the botulinum toxin affect on motor function changes over the time. The average age of the group was 52.43 years, with a range of 29–73, and it consisted of four men and three women. The average length of the stroke was 66.14 months, with a range of 10–160. Throughout the study, there were no serious adverse events or increases in pain related to the intervention.

**Table 1:** Participants’ demographic information, recorded in the screening session

ID	Gender	Age (years)	Paretic side	Stroke onset (months ago)
P01	Female	51	Right	10
P02	Male	33	Left	14
P03	Male	72	Right	144
P04	Female	52	Right	36
P05	Female	57	Left	75
P06	Male	73	Right	160
P07	Male	29	Right	24

### Primary outcomes

#### Recruitment and retention rates

Fifteen patients with stroke were invited to participate in the study, of which thirteen agreed to take part, resulting in a recruitment rate of 86.7%.

In total, eight stroke survivors were included in the study, and the retention rate was 87.5%, with seven participants successfully completing at least seven out of nine Tele BCI-FES home sessions. Only one participant withdrew from the study for unknown reasons.

#### Participation rate of patients in tele BCI-FES rehabilitation

The results of the study showed that the participation rate of the patients in the proposed Tele BCI-FES rehabilitation was excellent, as assessed by the PRPS. Six out of seven participants attended all nine Tele BCI-FES home sessions, while one participant (P03) attended seven Tele BCI-FES sessions due to illness. The mean PRPS score for the participants was 5.8 out of 6, which indicates a high level of participation [19]. This indicates that the patients were highly engaged in the telerehabilitation program.

#### Participants’ perception on adoption of the technology

**Fig. 6:**
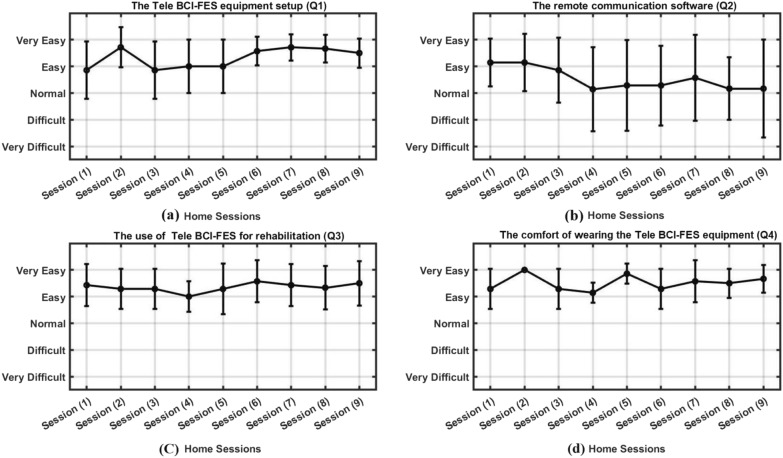
The line plot with error bars, presents the average responses obtained from quantitative interviews conducted with the seven participants during the nine Home-based Tele BCI-FES sessions. Subplots **a**–**d** are displaying the participants’ responses to the questions 1, 2, 3 and 4 respectively

Based on the feedback received throughout the experiment, participants generally had a positive experience with the ease of setting up and cleaning the BCI system. In the final qualitative interview, conducted in the final assessment session, one participant mentioned finding the equipment cleaning process tedious, while two others had no issues, and the remaining participants did not comment on it. The main complaint raised by three participants during the final interview was about the electrodes, which they found to be somewhat fiddly to use. While they managed to set up the system, they faced some difficulty inserting and removing the electrodes from the cap. This feedback highlighted a potential issue for improvement going forward. Interestingly, upon examining Fig. 6a, it becomes evident that by the final session, participants’ responses are centered between “easy” and “very easy” on average. This suggests that despite initial struggles, the majority of participants found the setup process to be moderately easy by the end of the study.

During the trial period, the effectiveness of the remote supervision provided was generally well received by participants. Overall, the majority of participants found the remote supervision to be effective in facilitating the sessions. However, it is worth noting that there were occasional issues with the remote communication software, particularly related to sound problems. These technical issues resulted in disruptions during some sessions, impacting the overall user experience. To mitigate the sound issues, research teams resorted to using phones for communication with the participants as an alternative method. This solution proved to be effective, enabling uninterrupted communication during the sessions. Despite this workaround, it was observed that the sessions affected by sound problems received lower scores, as indicated in Fig. 6b.

In addition to technical challenges, only two participants provided specific suggestions for improvement. They expressed concerns about the low volume of the beeps used during the sessions. One participant also mentioned that the initial video screen size was too small for their preference.

Based on the data presented in Fig. 6c, feedback about the ease of using the system for rehabilitation was generally positive. Participants found the instructions easy to follow and highly effective. One participant recommended adding some form of gamification, as they found the system monotonous over time, while two others appreciated the simplicity of the text, finding the lack of distractions beneficial.

When asked about the ease of wearing and comfort of the system, the primary issue raised by participants was the use of gel in the electrodes. One participant expressed being uncertain about using the system in the long term due to the gel, while two others stated they would be happy to use a few times per week, but a daily usage would be problematic due to the use of EEG gel. Cleaning out the gel took a while, especially for more disabled participants who needed assistance with showering. Some participants arranged their sessions for early morning or evening to allow time for cleaning. Our EEG cap was only provided in three sizes, i.e. small, medium and large, as these were the only options available from the manufacturer. As a result, two participants expressed concerns about the limited variation in cap sizes available, with one participant experiencing a slightly tight cap and two others facing a slightly loose cap. However most found the equipment comfortable to wear, with the exception of one participant who found the cap tight due to their hair growing over the course of the experiment (Fig. 6d).

Participants provided valuable feedback regarding potential improvements for the system, including implementing distinct beeps for different commands, incorporating a progress bar, and using dry electrodes. They also expressed a desire for more comprehensive information about brainwaves and BCI, as well as schematic diagrams to simplify the setup process.

Overall, both participants and caregivers showed motivation to continue using the Tele BCI-FES system, considering it worthwhile despite the additional setup requirements. Encouragingly, they also expressed a willingness to recommend it to other patients with stroke. However, certain aspects of the system, particularly the gel and the complexity of the setup process, should be addressed to enhance the overall user experience.

### Secondary outcomes (functional assessment)

**Fig. 7:**
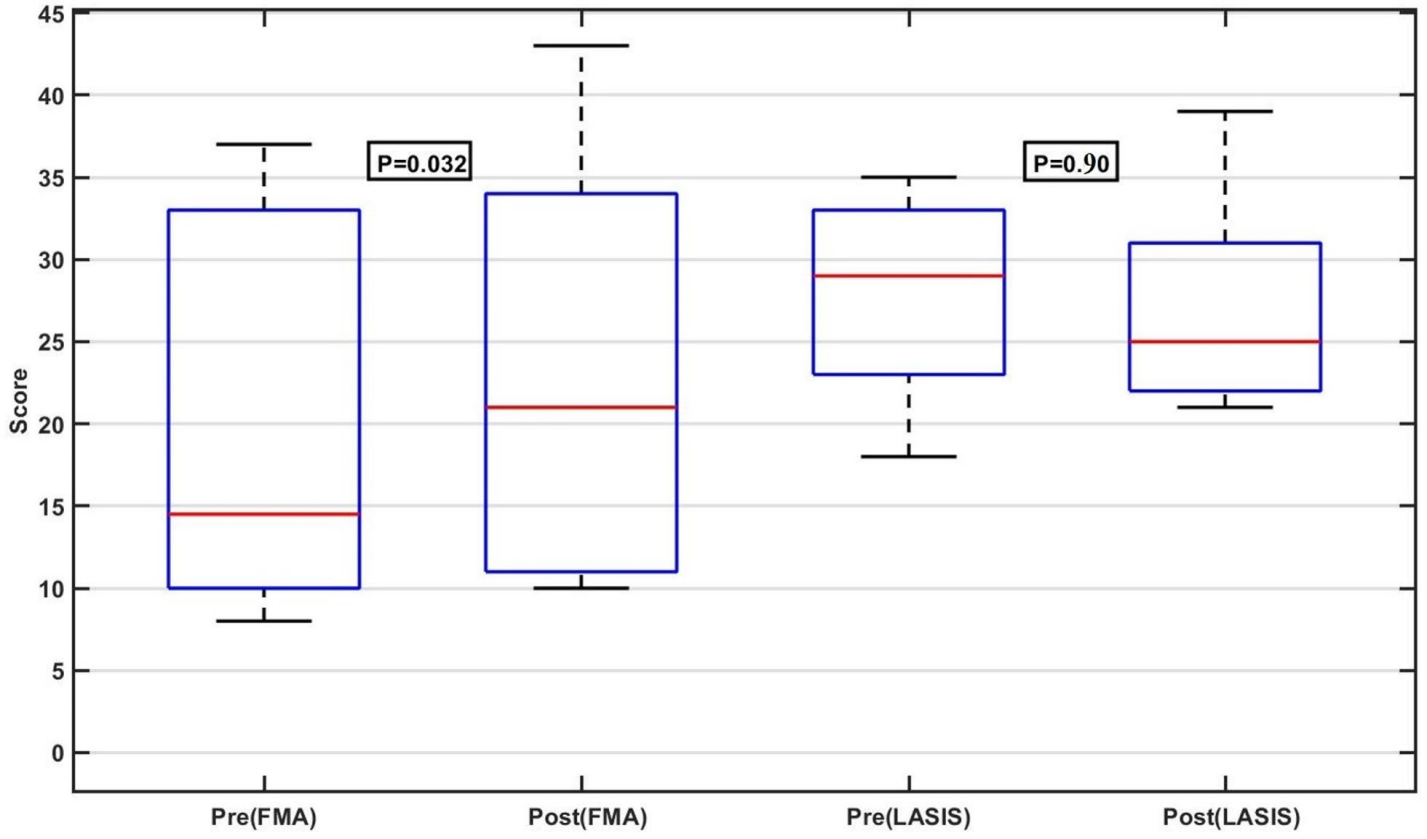
The box-plot shows average (pre-post) FMA_UE and LASIS scores of 6 patients with stroke

Table 2 and Fig. 7 show the FMA_UE and LASIS scores before and after the intervention for 6 out of 7 participants. One participant (P05) was not included in the functional assessment due to having received botulinum toxin treatment prior to the study. On average, there was a significant improvement in FMA_UE scores after intervention (mean = 23.33, p = 0.032) compared to pre-intervention (mean = 19.50). Hence, the differences between FMA_UE scores before and after the intervention was 3.83 points.

The observed improvement of 3.83 points in the FMA-UE score approaches the reported Minimal clinically important difference (MCID) of 4.25 points for patients with chronic stroke. Notably, two participants (P01 with +9 points and P03 with +6 points) exceeded the MCID, demonstrating clinically meaningful progress. This suggests that, even with a limited number of sessions, the intervention shows potential promise. Future studies could explore a greater number of sessions to potentially increase the effect size.

The high standard deviation of both pre and post measurements (± 12.44 and ± 12.97 respectively) suggests a large variability in the FMA_UE scores among participants. However, the statistical significance of the results (p = 0.032) highlights the overall positive effect of the Tele BCI-FES intervention on the FMA_UE score.

Based on the LASIS scores before and after the intervention, there were no consistent increases in spasticity across participants. As can be seen in Table 2, four out of six participants showed a reduction in LASIS scores, while two participants experienced an increase in spasticity. While the mean LASIS score slightly improved from 27.83 to 27.17 (p = 0.90), indicating a general trend toward reduced spasticity, the variability in response among participants underscores the importance of further research to explore factors that may influence variability in LASIS outcomes.

Notably, certain individuals (P01, P03, P04) experienced positive changes in their arm movement, including heightened awareness, enhanced stability, and increased mobility in the shoulder, elbow, and fingers. Moreover, the system enabled easier nail cutting, improved grip and release, and enhanced passive movement (P04).

It is important to emphasize that the primary focus of this study was to assess the acceptability and feasibility of the Tele BCI-FES rehabilitation approach. Given the limited number of sessions (9) provided in our study compared to other BCI rehabilitation studies which typically involved 18–20 sessions, a direct comparison in terms of motor function improvement is not viable. Furthermore, it should be noted that we did not conduct any follow-up assessments in the weeks following the conclusion of the intervention.

**Table 2:** Clinical scores for 6 participants

ID	FMA_UE		LASIS	
	Pre	Post	Pre	Post
P01	12	21	28	25
P02	17	21	35	31
P03	37	43	18	22
P04	8	10	33	25
P06	10	11	30	39
P07	33	34	23	21
Mean	19.50	23.33	27.83	27.17
± Std	12.44	12.97	6.37	6.76

P05 was administered botulinum toxin treatment prior to the study and was therefore excluded from the functional assessment

## Discussion

The present study aimed to investigate the feasibility and acceptability of a novel Tele BCI-FES system for upper limb rehabilitation in individuals with stroke. In this study, seven participants with chronic stroke completed a home-based Tele BCI-FES intervention. The results showed that the system is feasible and safe for use in individuals with stroke, with a high recruitment rate of 86.7% and a retention rate of 87.5%. Adherence rates to home-based rehabilitation therapies vary across studies. For example, adherence to traditional home exercise programs prescribed by physiotherapists has been reported to range between 50% and 80% [20]. In tele-rehabilitation, adherence rates can be comparable or even higher, as some studies suggest that these interventions maintain or improve patient adherence [21]. Factors such as patient engagement, design of the intervention, and level of support provided play critical roles in adherence rates. Compared to these benchmarks, the adherence rate observed in our study demonstrates strong patient engagement and supports the feasibility of Tele BCI-FES for home-based stroke rehabilitation. The participants’ feedback suggested that the system is generally acceptable. Moreover, the secondary outcome analysis showed that the Tele BCI-FES intervention resulted in a significant improvement in the FMA_UE score compared to the pre-intervention score. The findings of this study suggest that the proposed Tele BCI-FES system may be a promising tool for upper limb rehabilitation in individuals with stroke.

Interestingly, the high recruitment and retention rates suggest a strong interest in the use of the Tele BCI-FES system as a new rehabilitation tool. This is in line with previous studies that have shown a positive attitude towards the use of technology-assisted interventions and home-based training in stroke rehabilitation [22, 23]. The feedback from the participants suggested that the ease of setup for the BCI system was mixed, with some aspects being manageable while others were tedious or complex, particularly in regards to connecting the electrode cables. However, as seen in Fig. 6, participants reported an increased ease of use and efficiency in setting up the system with each subsequent session. Having said that, these findings highlight the importance of user-centered design in the development of such technologies, with a particular focus on ensuring ease of use and minimizing the burden on the user [24].

When considering the utilization of the Tele BCI-FES system for rehabilitation purposes, participants’ feedback highlighted concerns regarding the gel used in the electrodes. In order to address this issue, dry electrodes present themselves as a potentially convenient alternative. Unlike wet electrodes, dry electrodes eliminate the need for conductive gel or saline solution, simplifying the application process and minimizing messiness. However, it is worth noting that dry electrodes may yield lower quality signals compared to wet electrodes, potentially impacting the accuracy of collected data [25, 26]. Additionally, certain designs of dry electrodes, characterized by spiky textures, have been associated with reported pain and discomfort when used for extended periods of time [27].

In terms of functional assessment, one participant (P05) was excluded due to receiving botulinum toxin a few weeks prior to the study. Botulinum toxin treatment can reduce spasticity, which may help improve motor function for a few weeks [28]. Therefore, this improvement in motor function could have affected the results of the assessment. This demonstrates the importance of careful participant selection and consideration of confounding factors when conducting research.

The functional assessment analysis of 6 participants showed a significant improvement in the FMA_UE scores after the Tele BCI-FES intervention, with an average increase of 3.83 points. This suggests that, even with a limited number of sessions, the intervention shows potential promise. Future studies could explore a greater number of sessions to potentially increase the effect size. In addition, the present study demonstrated that the Tele BCI-FES system has the potential to improve motor function in chronic and patients with severe stroke, even several years after the stroke (see Tables 1 and 2). Importantly, some participants reported some improvements in their arm movement, with increased movement in the shoulder, elbow, and fingers. However, the large variability in FMA-UE scores among participants highlights the importance of identifying potential factors that may influence the BCI treatment responses. Future studies should investigate the optimal parameters for Tele BCI-FES interventions, including the intensity, frequency, and duration of the intervention [29].

It is worth noting that the Tele BCI-FES intervention in our study had a relatively short duration, consisting of only nine sessions, which is shorter compared to other lab-based BCI studies such as the study by Sebastian et al. [30] and Miao et al. [31]. Specifically, in the study by Sebastian et al., patients with stroke received 25 sessions of BCI-FES intervention in a laboratory setting. Despite the remote and brief intervention period in our study, we obtained promising results, suggesting that even a limited amount of Tele BCI-FES intervention can have a positive impact on upper limb stroke rehabilitation in a home setting. However, further research is needed to determine the optimal duration and frequency of Tele BCI-FES intervention for patients with stroke in a home setting. This information could help to guide the development of more effective and efficient rehabilitation protocols, and enhancing patient outcomes.

Overall, our findings add to the body of evidence supporting the growing trend towards home-based medical care by demonstrating the feasibility and acceptability of Tele BCI-FES for upper limb stroke rehabilitation in a home setting [32–34]. These results suggest that home-based care options have the potential to improve outcomes for patients with stroke and highlight the need for continued research in this area. By providing access to effective rehabilitation interventions in a familiar and comfortable environment, home-based care may offer a promising alternative to traditional clinic-based rehabilitation, particularly for patients with geographical or mobility constraints.

## Limitations and improvements

The study’s findings are limited by the small sample size, which means that they cannot be widely applied. Further research with larger sample sizes and longer intervention period is necessary to confirm the effectiveness of Tele BCI-FES in improving upper extremity motor function in patients with stroke. The set-up process for the Tele BCI-FES system could also be improved to be more user-friendly for less technically-minded participants. Additional labels or instructions could be provided to help participants navigate the system more easily. Furthermore, an initial in-person session at the participant’s house to help set up the equipment and show how it works could be a useful improvement to ensure a smooth and comfortable experience for the participants during the study. The use of none-gel EEG electrodes can be considered in future studies to ensure participants’ convenience. However, it’s also important to ensure that the electrodes are effective and comfortable for the user to wear.

During the study, it was found that the audio quality using third-party video conferencing software between the research team and participants was not always effective. As a result, the research team sometimes had to resort to using phone calls to communicate with participants. Additionally, a few participants encountered challenges when trying to open the webcam and audio during home sessions due to the small size of the icon. Therefore, it is recommended that alternative video conferencing software and methods be explored in future studies.

## Conclusion

In summary, the present study offers evidence supporting the feasibility and acceptability of the proposed Tele BCI-FES system for upper limb rehabilitation in individuals with chronic stroke. The high recruitment rate emphasizes the patients’ eagerness for a new rehabilitation approach. Despite suggestions for future improvements, the overall retention rates, ease of use, and positive feedback from participants indicate a strong acceptance of this device. The noteworthy improvement in FMA_UE scores underscores the potential of the Tele BCI-FES system to enhance motor function in chronic and patients with severe stroke, even years after the stroke occurred. Nevertheless, further research is required to fine-tune intervention parameters and assess the effectiveness of this technology in larger sample sizes and longer intervention periods.

In conclusion, the findings offer promising evidence for the role of Tele BCI-FES as a valuable tool in stroke rehabilitation.

## Data Availability

The datasets generated and/or analyzed during the current study are not publicly available due to the requirements of the ethics approval agency, but are available only for research purposes upon request and after obtaining the required approval.
